# Knowledge, attitudes, and practices of Crimean Congo hemorrhagic fever among livestock value chain actors in Kagadi district, Uganda

**DOI:** 10.1371/journal.pntd.0011107

**Published:** 2023-02-02

**Authors:** Dreck Ayebare, Muzafalu Menya, Alex Mulyowa, Adam Muhwezi, Robert Tweyongyere, Stella A. Atim

**Affiliations:** 1 Royal (Dick) School of Veterinary Studies, University of Edinburgh, Edinburgh, United Kingdom; 2 Ministry of Agriculture, Animal Industry and Fisheries, Entebbe, Uganda; 3 Makerere University, Kampala, Uganda; Fort Collins, UNITED STATES

## Abstract

**Background:**

Crimean Congo hemorrhagic fever (CCHF) is a zoonotic tick-borne disease with an increasing number of outbreaks among communities in Uganda. Following the disease outbreak in the western district of Kagadi on 20^th^ February 2020, a KAP survey was conducted to identify knowledge gaps and at-risk behaviors related to the disease among livestock value chain actors.

**Methods:**

A household survey using a semi-structured questionnaire was conducted in 399 households in the two sub counties of Bwikara and Ruteete, Kagadi district. A focus group discussion with members of the community was conducted as well as key informant interviews with at-risk individuals.

Descriptive and inferential analysis was performed using STATA version 13 (Statacorp Texas; USA). Comparative analysis of the data from the two sub counties was also performed using cross tabulations in STATA, between each independent variable and the subcounty variable. The descriptive and comparative statistics used were minimum, mean and maximum values, standard deviations, frequencies, percentages, chi square values and t-statistics. A chi-square test was then employed on each tabulation, to determine whether there was an association between the two categorical variables or not. The test was set at an alpha level of 0.05, and where the p-value was less than or equal to the alpha value, we concluded that the 2 variables were associated.

**Results:**

Although majority of the respondents believed in the existence of the disease, only 12.8% had knowledge of prevention measures against CCHF. 67.2% of the respondents reported regular interaction with ticks during routine farm operations and they employed tick control measures on their farms. Although the respondents believe the disease is fatal, almost all of them (99%) would welcome a CCHF survivor back into the community. 95.2% of the respondents actively attended to animals but only 25.8% participated in slaughtering animals.

Qualitatively, the technical informants had knowledge about CCHF but non technical informants hardly knew about the disease. Limited funding appropriated for local governments, as well as limited engagement in One health activities were some of the barriers highlighted towards the infection prevention and control activities.

Most of the focus group discussion participants knew about the disease, but lacked knowledge on its transmission and prevention. Limited access to personal protective equipment and high exposure to tick-prevalent areas when slaughtering and grazing animals respectively, were the major challenges highlighted.

**Conclusion:**

Knowledge on CCHF among majority of the respondents was poor. There is a need for educational programs to increase awareness of CCHF in communities. This awareness should be done by both the community leaders and technical people to ensure the community receives enough knowledge on how to prevent and control the disease. To ensure effectiveness of these programs a One health approach should be adopted to implement prevention and control strategies.

## Author summary

Crimean Congo hemorrhagic fever causes widespread outbreaks in areas where the principal tick vector is found. In Uganda, several outbreaks have been reported in recent years and this is attributed to silent circulation of the causative agent in ticks and animals which later spills to humans. Unfortunately, there is no vaccine for the disease and the only available treatment option is primarily supportive. Data to inform design of interventions that can prevent the emergence and spread of the disease in high-risk areas is limited. We, therefore, decided to investigate, knowledge, attitudes, practices and risk of the disease in Kagadi district where an outbreak was confirmed in 2020. We found low levels of knowledge on how to prevent the disease although most people in this area participated in activities that put them at a risk of contracting the disease. Funding to support prevention and control of the disease in communities is limited. Additionally, there was poor multi-disciplinary coordination during response to the disease outbreak. This study, therefore, recommends an educational program to sensitize communities on the disease using a multi-disciplinary approach where all related sectors are involved in prevention and control activities.

## Introduction

Crimean Congo hemorrhagic fever (CCHF) is a zoonotic tick-borne viral infection, geographically widespread with many cases occurring in sub-Saharan Africa. It is caused by Crimean Congo hemorrhagic fever virus, RNA *Orthonairovirus* of the *Nairoviridae* family [1]

This disease is transmitted by a bite from an infected *ixodid* tick or crushing an infected tick with bare skin. In Uganda, ixodid ticks, including the Hyalomma ticks (which is the principal reservoir of the virus), have been reported [2] Transmission is also reported to occur through contact with infected animal blood tissues and ingesting unpasteurized milk. In health care settings, human to human infection occurs through exposure to blood or bodily fluids of infected individuals.

Although CCHF affects animals, they don’t show any clinical signs. However, about 20% of humans infected with the virus exhibit clinically fatal illness [3]. In humans, the disease first presents as a feverish illness and then rapidly progresses to a hemorrhagic syndrome. This results in multiorgan failure and death in severe cases. It’s not surprising that the fatality rate has been reported to be as high as 60% in some regions, and therefore, it is a notifiable disease in many countries. Although there is no published report indicating the economic impact of CCHF globally, other closely related hemorrhagic fevers are reported to cause huge losses to the economy both directly and indirectly. For example, a dengue hemorrhagic fever outbreak cost Brazil an estimated US$ 1,228 million in 2013 [4] and in 2007, a Rift valley fever outbreak in Kenya resulted in US$ 32 million economic loss [5]. In Uganda, there have been epidemic outbreaks in recent years with the last epidemic reported in January 2020 in Kagadi district. Studies have indicated that the disease mainly affects people involved in the livestock sector, particularly slaughterhouse workers, veterinarians, and livestock keepers [6]. The outbreaks are expected to continue, especially in livestock communities because of ineffective tick control resulting in propagation of *Hyalomma* tick populations. There is no vaccine for the disease and the available therapeutic interventions are restricted to treating symptoms.

Beyond the detected cases, the knowledge, attitudes, and practices of at-risk individuals in the areas reporting the CCHF outbreaks are not well understood. Thus, data to inform public health interventions in the affected communities is limited. Although a limited number of “knowledge, attitudes and practices” (KAP) studies have been conducted in Asia, there are no published CCHF studies in Africa and Uganda specifically, although the disease continues to pose a significant public health threat to the region.

This “knowledge, attitudes and practices” (KAP)/ risk study aimed at providing baseline data for further assessments and help in strengthening the behavioral and communication interventions on the response of CCHF and other related emerging infections. This study identified knowledge gaps and at-risk behaviors related to CCHF.

Specifically, this study set out to:

Determine the level of knowledge, attitudes, and awareness on CCHF in Kagadi district.

Identify risks for transmission of CCHF in Kagadi district.

Determine the current practices for prevention and control of CCHF in Kagadi district.

## Methodology

### Ethics statement

This study was undertaken as part of the Arboviral infection study (AFI) approved by the ethics review committee at the College of Veterinary Medicine, Animal Resources and Biosecurity of Makerere University, Kampala, Uganda (Reference Number: SVARREC/20/20l8) and by the Uganda National Council for Science and Technology (Reference Number: HS 2485). Informed written consent was obtained from all study participants before they were enrolled into the study.

### Geography, climate, and economic activities in the area of study

**Fig 1:**
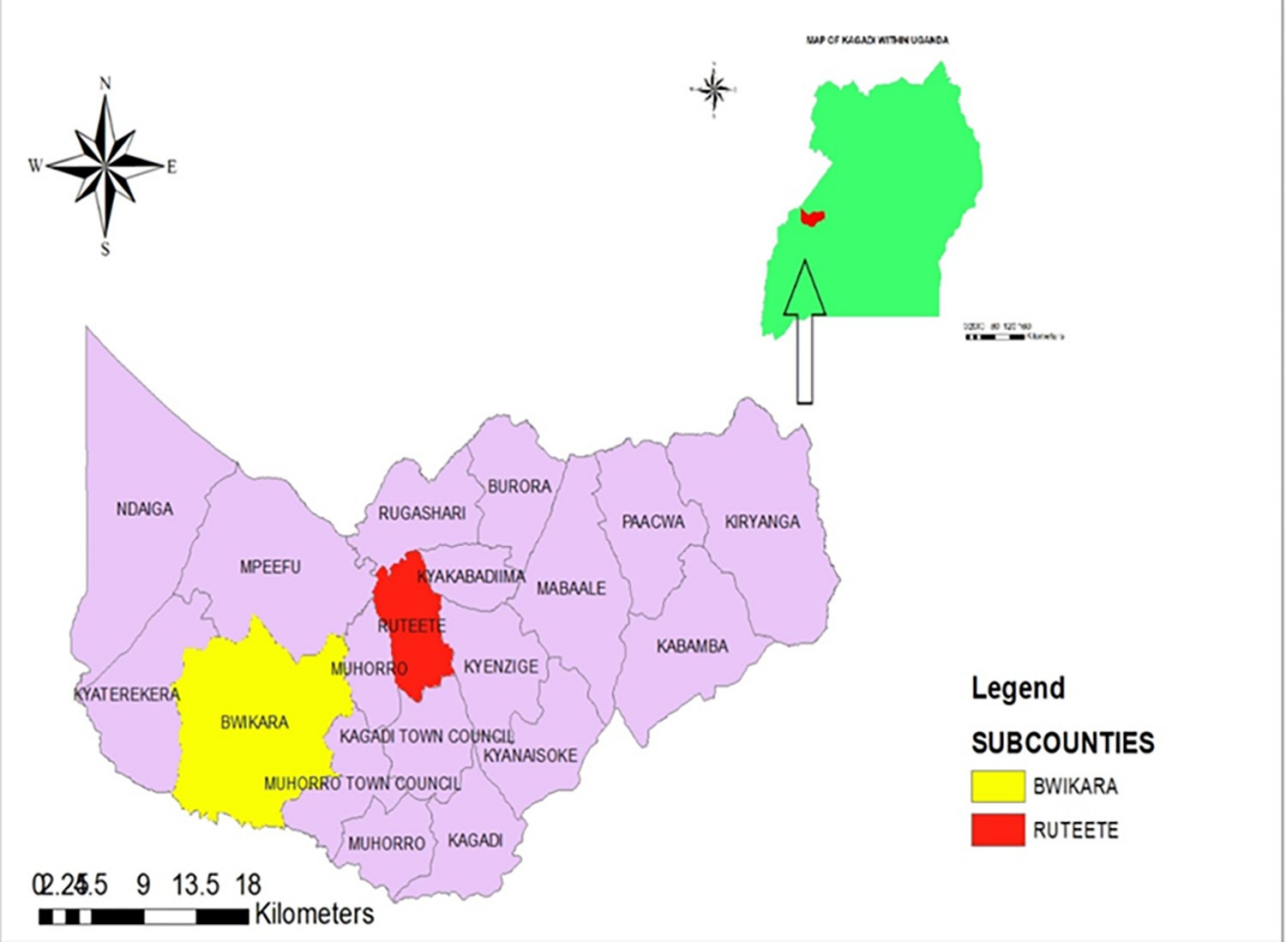
A map of Kagadi district showing Bwikara and Ruteete sub counties. This map was drawn using data sets downloaded from the Uganda districts Shapefiles website https://data.unhcr.org.

This study was conducted in Bwikara and Ruteete sub counties in Kagadi district **(Fig 1)**. It is bordered by Ntoroko district to the west, Hoima to the north, Kibaale to the east and Kyenjojo to the south. The district is estimated to have a population of 459,700 people and 69% of the inhabitants live in rural communities where the main economic activity is farming [7]. Agriculture is the main economic activity in this households with most households growing crops and rearing animals.

This district was selected for this study because of the CCHF outbreak that occurred in 2020 [8]. It was reported that CCHF was confirmed in a 23-year-old male lumberjack from the western district of Kagadi after a sample was collected and analyzed at Uganda Virus Research Institute (UVRI) on 21^st^ January 2020. He had developed fever on 7th January 2020 and self-medicated for malaria without any response. There was no history of contact with meat. The fever progressed into generalized body weakness, abdominal pain and two weeks later, he started vomiting and urinating blood as well as bleeding from the nose. He was then rushed to Kagadi hospital, put under isolation and UVRI was informed. Fortunately, the patient was treated, and he recovered.

### Research design and data collection

This study employed a cross-sectional design (between July and December 2021) to collect information from stakeholders along the livestock value chain in Kagadi district. A semi-structured questionnaire was designed by the researchers and used to collect data from farmers within the sub counties of Bwikara and Ruteete. Key informant interviews were conducted to collect data from a policy maker (district production officer), veterinarians (official veterinarians for Bwikara and Ruteete sub counties), a laboratory worker, a slaughterhouse worker, a retail meat trader, and a cattle trader.

Additionally, a focus group discussion (FGD) was conducted in July 2021 with participants from Ruteete and Bwikara sub counties. The FGD consisted of 8 participants from the 2 sub counties, and it collected data on knowledge practices, attitudes as well as risks for Crimean Congo hemorrhagic fever emergence and spread.

In this study, the population consisted of all livestock keeping households in Kagadi district. The sample size was calculated based on an alpha of 0.05 and beta of 0.2 using the formula N = (z2 × s 2)/d2, where the z value is 1.96 at a confidence interval of 0.95%, s shows the standard deviation equal to 0.05, and d represents the extent of accuracy considered as 0.06. The obtained sample size was 267 farms. However, a total of 399 households were recruited to cater for any errors in data collected since the study was conducted during the pandemic when training of research assistants was prohibited and there was a number of challenges anticipated during the data collection exercise. A questionnaire tool uploaded onto ODK software was used to collect data from livestock farmers.

## Results

### Socio-demographic characteristics of the livestock value chain actors

Table 1 presents the sociodemographic characteristics of the respondents. About a half, 50.6%, of these were female; and were in the age category of 31–40 years (39.1%). Less than half, 42.1%, of the respondents had a primary level of education, whose major source of livelihood was farming, at 94.0%.

**Table 1:** Socio-demographic characteristics.

Variable	Frequencies (n = 399)	Percentages (%)
***Age category (years)***		
<18	0	0
18–30	76	19.1
31–40	156	39.1
41–50	115	28.8
51–60	41	10.3
>60	11	2.8
***Household numbers***		
<5	58	14.6
5 to 8	54	13.6
9 to 12	56	14.1
Above 12	230	57.8
***Gender***		
Female	202	50.6
Male	197	49.4
***Education Level***		
No formal education	137	34.4
Primary	168	42.1
Secondary (A-level)	3	0.8
Secondary (O-level)	68	17
Tertiary	23	5.8
***Parishes***		
Kanyangoma	102	25.6
Kihayura	100	25.1
Kisuura	130	32.6
Nyamasa	67	16.8
***Sub-county***		
Bwikara	197	49.4
Ruteete	202	50.6
***Major source of livelihood***		
Business	19	4.8
Farming	375	94
Salary	5	1.3

### Diseases in the herd in the past one year

**Fig 2:**
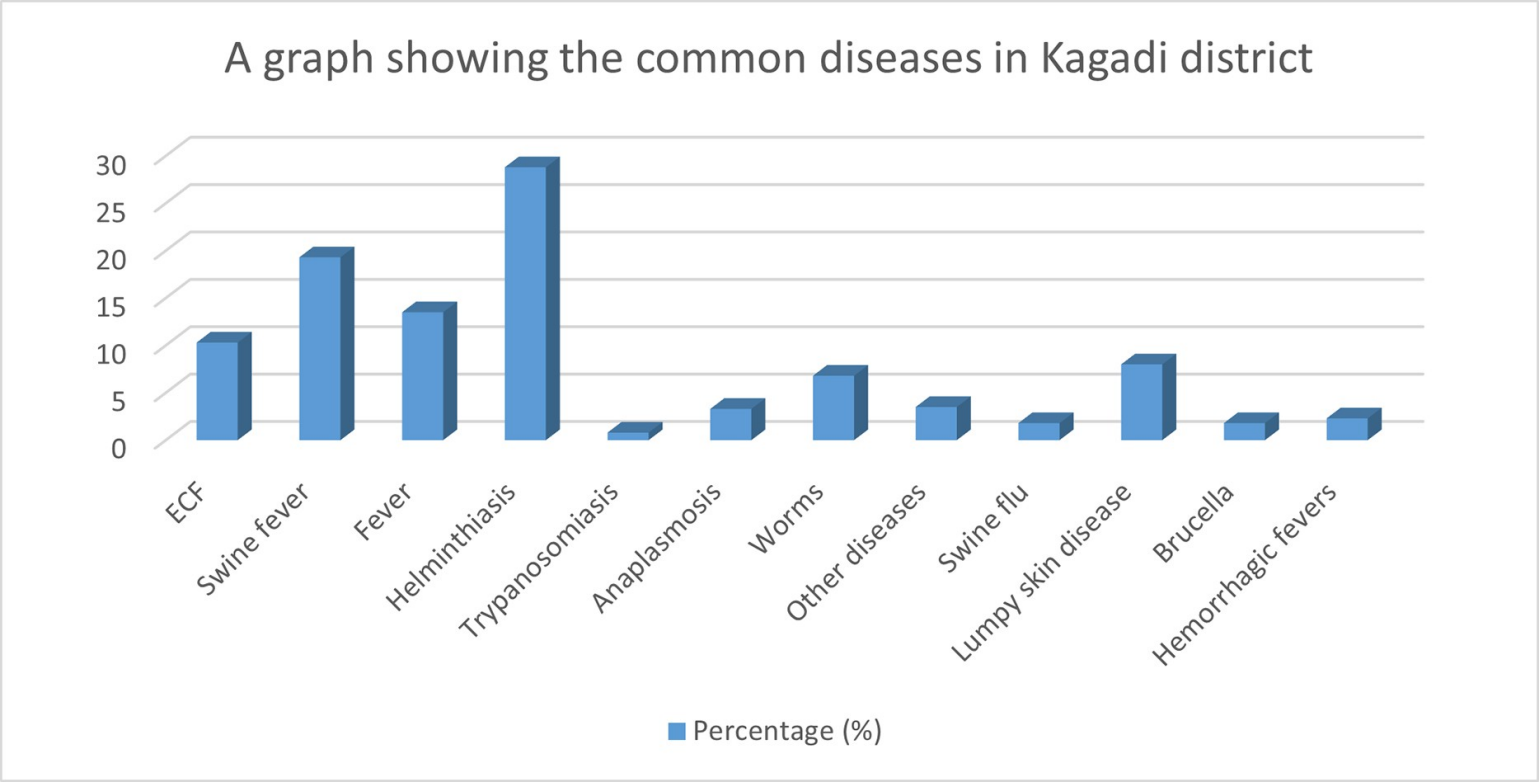
A bar graph showing the common diseases in the study area.

Fig 2 presents diseases that were observed in the herd in the last one year. The major diseases that affected the animals in the past year were helminthiasis (28.8%) and African swine fever (19.3%). The major animals that had been affected were pigs, 57.6%, and goats (27.4%). These diseases occurred mostly in the rainy season (61.7%) in the past year.

### Animals that are most affected by diseases

**Fig 3:**
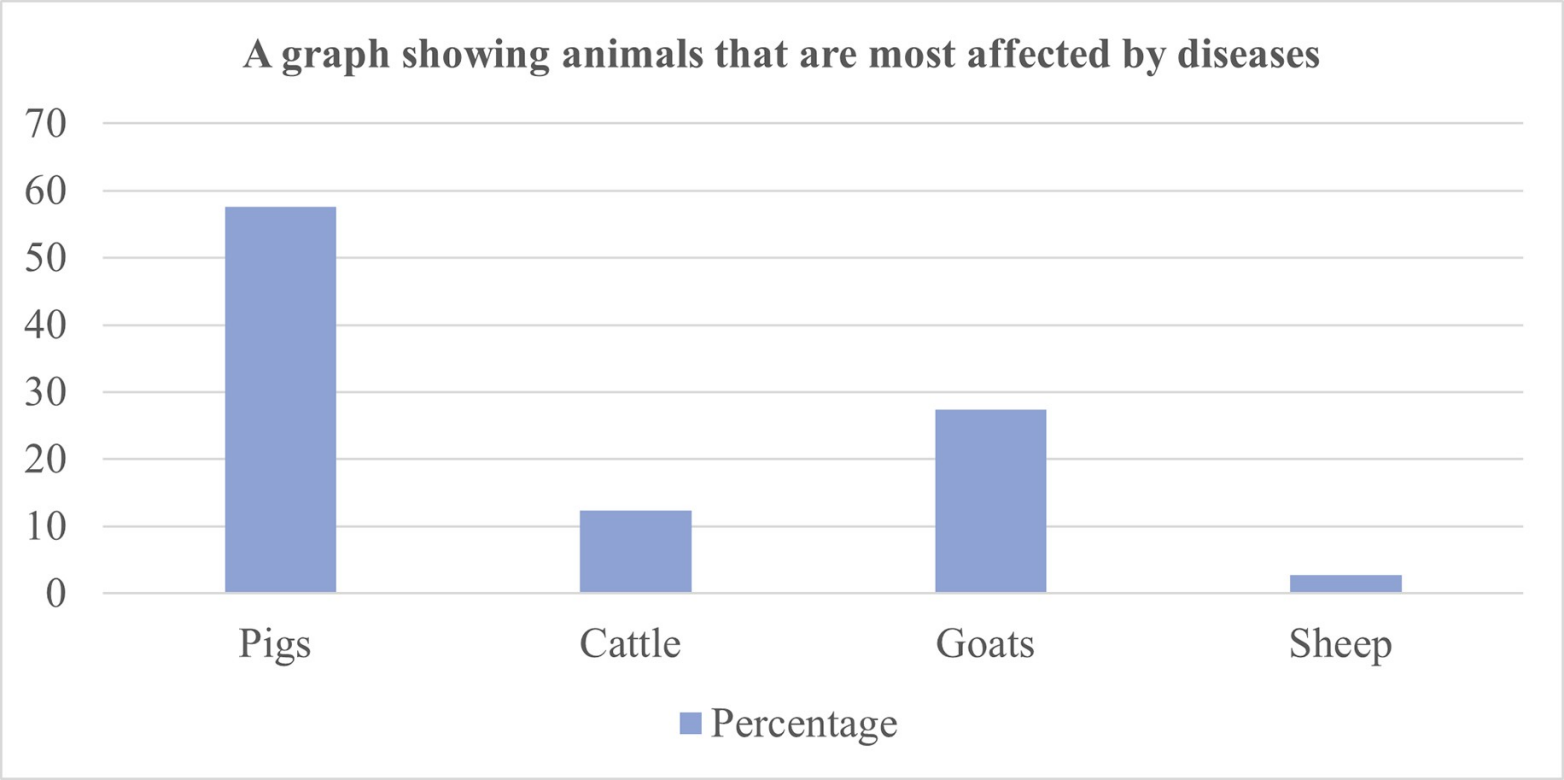
A bar graph showing animals most affected by diseases in the study area.

Fig 3 shows animal species that are most affected by diseases. Pigs are the most affected animals, followed by cattle. Sheep are rarely affected by diseases.

### Average number of animals kept per household

**Fig 4:**
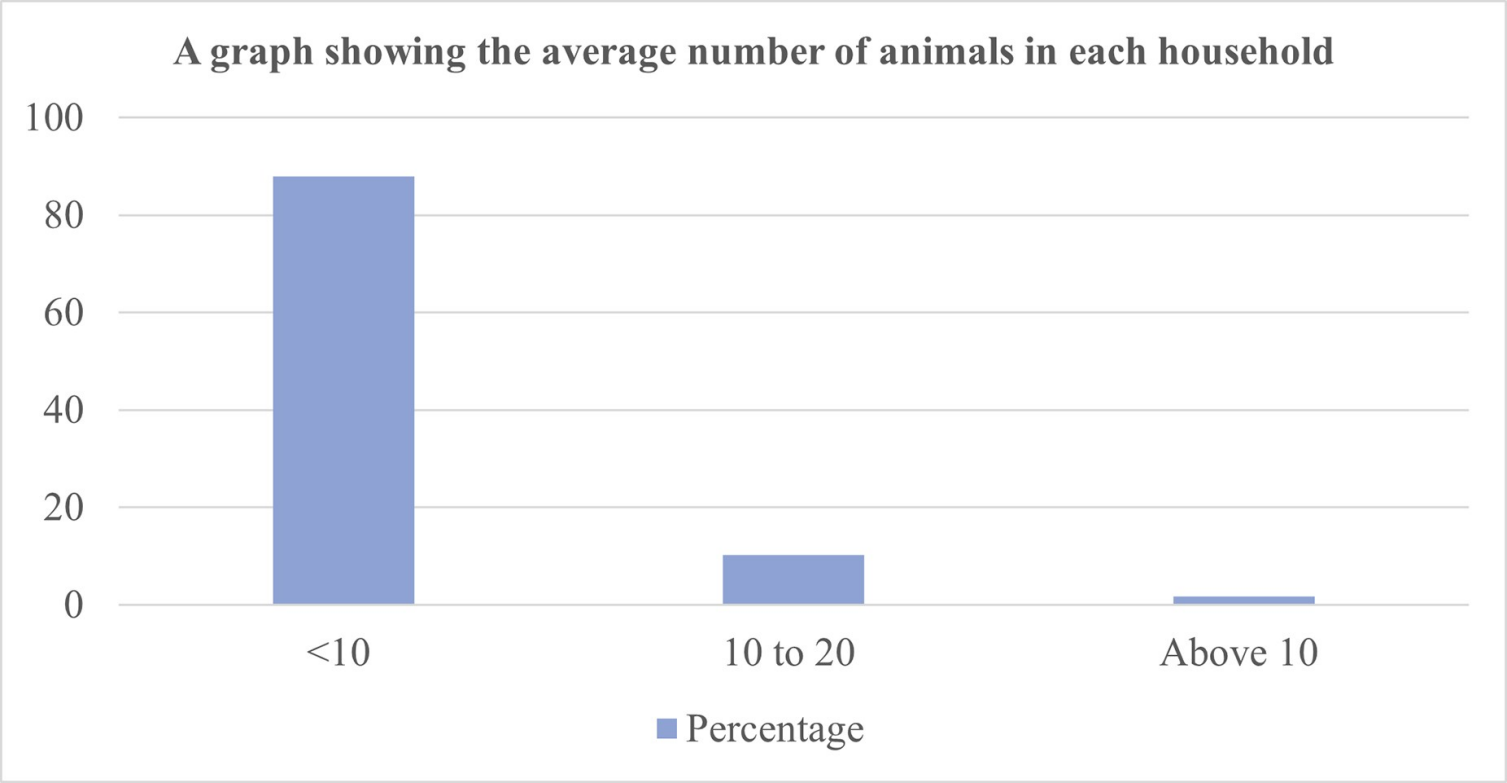
A bar graph showing the average number of animals per household.

Fig 4 indicates the average number of animals kept in each household in the study area. Most of the households kept less than 10 animals while less than 2% of the households reared more than 10 animals.

### Distribution of diseases between seasons

**Fig 5:**
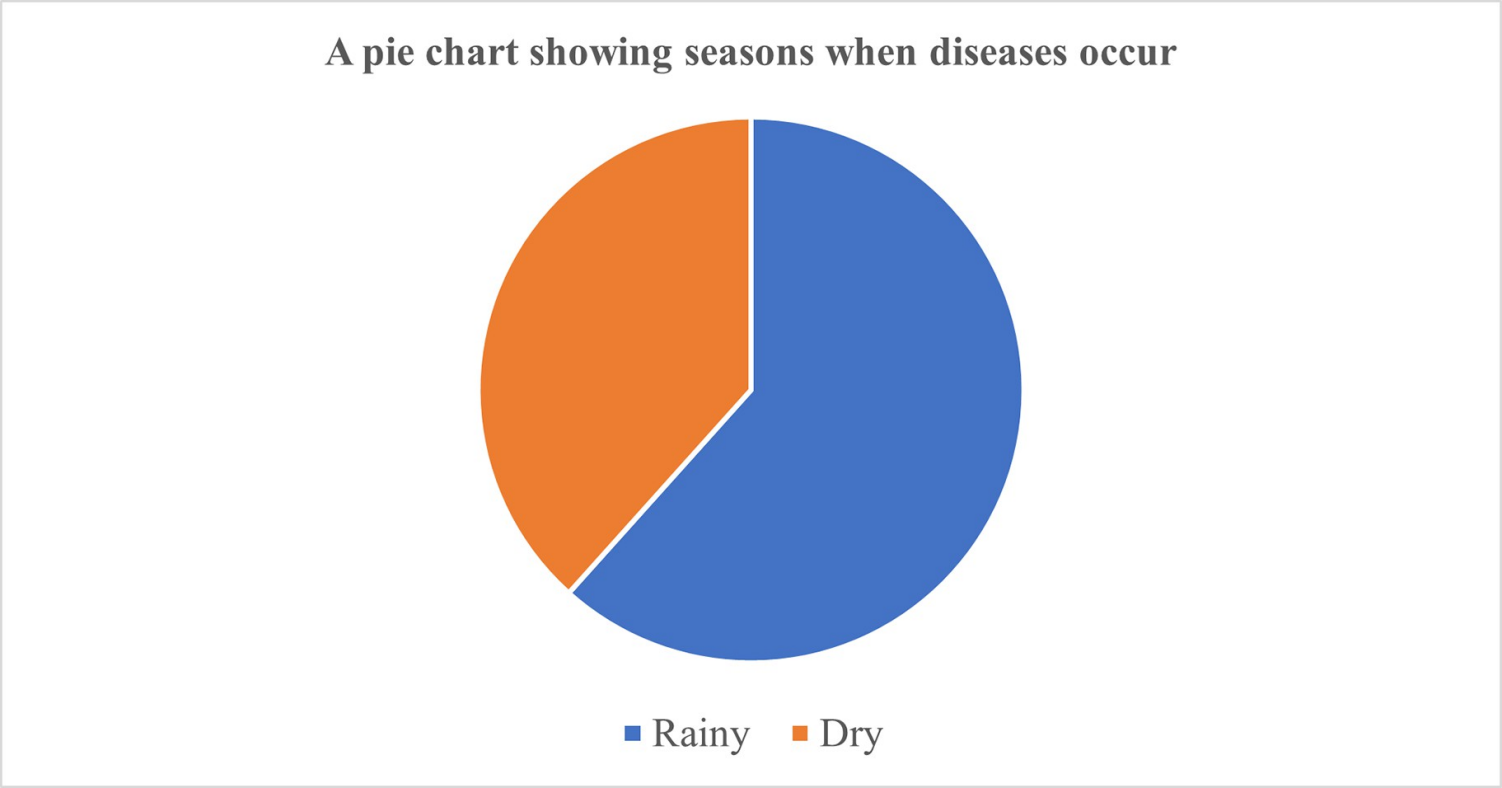
A pie chart showing distribution of diseases between seasons.

Fig 5 shows that most of the diseases in livestock during the rainy season compared to the dry season.

### Management of diseased animals

Table 2 shows that 65.2% of the respondents treated the animals themselves when they became diseased, followed by consultation of a private veterinarian (35.3%). Consultation of non-governmental organizations (NGOs) and traditional healers were the least reported measures on management of disease among the animals.

**Table 2:** Measures taken to manage diseases in animals.

Variable	Frequency (n = 399)	Percentage (%)
***Treat sick animals myself***		
No	139	34.8
Yes	260	65.2
***Consult traditional healers***		
No	399	100
Yes	0	0
***Consult community animal health worker***		
No	380	95.2
Yes	19	4.8
***Consult private veterinarian***		
No	258	64.6
Yes	141	35.3
***Consult with government worker***		
No	337	84.5
Yes	62	15.5
***Consult NGO***		
No	398	84.5
Yes	1	0.3

### Knowledge and attitudes of livestock value chain actors on CCHF

Table 3 shows that 58.9% of the respondents had poor knowledge regarding CCHF. Only 37.8% of the respondents knew the clinical signs of CCHF, of which fever (35.8%) was the most reported sign. Of those respondents that reported knowing the transmission mode, 30.6% reported blood and other tissues as the most common transmission mode. Additionally, 87.2% of the respondents had no knowledge of prevention measures against CCHF.

**Table 3:** Knowledge and attitude of value chain actors towards CCHF.

Variable	Frequency(n = 399)	Percentage (%)
***Heard of CCHF***		
No	217	54.4
Yes	182	45.6
***Source of information about CCHF***		
Community leaders	95	52.2
Health worker	10	5.9
Radio	77	42.3
***Knowledge of signs and symptoms***		
No	248	62.2
Yes	151	37.8
***Signs and symptoms***		
Bleeding	59	14.8
Fever	143	35.8
Diarrhea	39	9.8
***Knowledge of contact person***		
No	213	53.8
Yes	186	46.6
***Interaction with CCHF survivor***		
No	188	47.1
Yes	211	52.9
***Welcome CCHF survivor back into community***		
No	4	1
Yes	395	99
***Belief in existence of CCHF***		
No	39	9.8
Yes	360	90.2
***Transmission of CCHF***		
No	256	64.2
Yes	143	35.8
***Transmission mode***		
Tick bite	41	10.3
Blood and other tissues	122	30.6
Other	2	0.5
***Protection from CCHF***		
Avoid contact with other animals	215	53.9
Avoid contact with ticks	143	35.8
Others	41	10.3
***Risk perception***		
No	316	79.2
Yes	83	20.8
***Knowledge of prevention measures***		
No	348	87.2
Yes	51	12.8
***Knowledge score 3*.*0(±2*.*1) (mean(±SD)***		
Low	235	58.9
High	164	41.1

Regarding attitudes, 90.2% of the respondents believed in the existence of CCHF; and similarly, 99.0% would welcome a CCHF survivor back into the community. Risk perception among respondents towards CCHF was 20.8%, and 52.9% of the respondents would have an interaction with a CCHF survivor.

### Epidemiological risks related to CCHF among livestock value chain actors

67.2% of the respondents had had an interaction with ticks during the course of their work. 75.2% of the respondents reported having bushes around their farms. Respondents that reported to be drinking raw milk and eating raw meat were very few, at 1% and 0.5% respectively. The results are presented in Table 4.

**Table 4:** Epidemiological risks related to CCHF.

Variable	Frequency (n = 399)	Percentage (%)
***Had any interaction with ticks during work***		
No	131	32.8
Yes	268	67.2
***Drank raw milk***		
No	395	99
Yes	4	1
***Ate raw meat***		
No	397	99.5
Yes	2	0.5
***Are there bushes around the farm***		
No	99	24.8
Yes	300	75.2

### Proposing risks related to CCHF among livestock value chain actors

Table 5 indicates the risks related to the disease among different livestock value chain actors in the area. 95.2% of the respondents were actively involved in attending to animals; but only 25.8% were involved in slaughter or assisted with butchering livestock. 52.9% of the respondents also reported having had vermin on their farms.

**Table 5:** Risks related to CCHF amongst livestock value chain actors.

Variables	Frequency (n = 399)	Percentage (%)
***Are there wild animals like antelopes*, *warthogs that come into your farm***		
No	271	67.9
Yes	128	32.1
***Have any vermin in the farms***		
No	188	47.1
Yes	211	52.9
***Hire manual laborers to clear open farmland***		
No	358	89.7
Yes	41	10.3
***Slaughter/ assists with butchering livestock***		
No	296	74.2
Yes	103	25.8
***Actively involved in attending to animals***		
No	19	4.8
Yes	380	95.2

### Practices related to prevention and control of CCHF among livestock value chain actors

Table 6 shows practices related to CCHF prevention and control in the study area. 41% of the respondents used protective clothing as a preventive measure, and of these, 93.4% reported to be wearing it sometimes. Of those that reported to have observed ticks in their farms in the last 6 months, 69.4% controlled them by spraying/dipping their animals. Tick control methods were mainly applied once a month. For those that experienced symptoms of CCHF, 91.5% sought care from a hospital/healthcare center.

**Table 6:** Practices related to prevention and control of CCHF.

Variable	Frequency (n = 399)	Percentage (%)
***Use protective clothing***		
No	232	58.2
Yes	167	41.9
***Treat clothing with repellent***		
No	399	100
Yes	0	0
***Use insect repellent on self***		
No	395	99
Yes	4	1
***Often use protective clothing to protect from ticks/CCHF***		
Sometimes	156	93.4
Always	5	3
Never	6	3.6
***Often use pesticide in the environment***		
Sometimes	73	65.8
Always	29	26.1
Never	9	8.1
***Often avoid woody/rural areas***		
Sometimes	89	89
Always	4	4
Never	7	7
***Sight of ticks in the farm for the last 6 months***		
No	141	35.3
Yes	258	64.7
***Control methods***		
Spraying/dipping	179	69.4
Hand picking	74	28.7
Bush burning	3	1.2
Others	13	5
***Frequency of application of the ticks control methods above***		
Once in 2 weeks	72	27.9
Once a week	75	29.1
Once a month	95	36.8
Others	16	6.2
***Tick control in other animals***		
Goats	64	62.1
Sheep	14	13.6
Pigs	69	67
Dogs	13	12.6
***Protective clothing while spraying animals with acaricides***		
No	146	56.6
Yes	112	43.4
***Care sought if any***		
Go to a hospital/healthcare	236	91.5
Go to a local healer	2	0.8
Try local pharmacy	19	7.4
Stay at home	1	0.4

### Qualitative results

#### Key informant interviews

Six respondents were interviewed in the key informant interviews (KII). One focus group discussion was conducted, and it involved eight participants (five males, three females), the researcher and a research assistant who took notes and recorded the discussion. Consent was obtained to take recordings and photos of participants.

The respondents in the KII were the area veterinarians of Ruteete and Bwikara sub counties, a cattle trader, a slaughterhouse worker, a laboratory technician at Kagadi hospital, the district production officer (DPO) and a retail meat seller while the FGD participants were farmers.

Results from the focus group discussion and key informant interviews were combined and transcribed to generate transcripts that were manually coded into key themes from which several key sub-themes were identified. Through thematic content analysis, codes were manually grouped to form three categories, which emerged into key themes namely, knowledge, attitudes, and awareness as theme 1, practices for prevention and control as theme 2 and epidemiological risk factors as theme 3. These themes represented majority of the responses and were presented as texts and narrations. This is presented in Table 7 below.

**Table 7:** Major themes generated from KIIs and FGD.

Major result themes	
Theme 1	Participants knew about CCHF and stated that it can be transmitted from one person to another. They reported having heard about it on the radio after the 2020 outbreak. The symptoms highlighted include vomiting and bleeding from the mouths and nose. They considered it to be a dangerous disease.
Theme 2	Participants noted that they would seek medical care if they experienced symptoms of CCHF. All the participants attended to animals routinely but only three of these wore gumboots. 5 of the 8 FGD respondents admitted that they rarely control ticks on their farms. This was attributed to high costs of acaricides. The 3 FGD participants who control ticks spray their animals with acaricides monthly but other animals including pigs, sheep and goats are not sprayed with acaricides.
Theme 3	All the participants believe that they are at risk of getting infected with the virus since they are always in close contact with ticks and animals. 5 FGD participants admitted that they always crush ticks with bare hands. 5 of the 8 FGD participants admitted to eating half-cooked meat, especially offals. Participants admitted to slaughtering animals at home, especially during festive seasons or when they get a visitor. Of these, none reported using personal protective equipment like gloves and gumboots to avoid contact with bodily fluids from animals

#### Knowledge, attitudes, and awareness

The laboratory technician, veterinarians and the DPO knew about the disease, its signs, and symptoms as well as its mode of transmission. The signs and symptoms mentioned were fever, vomiting and bleeding from the mouth and nose. These respondents also highlighted that CCHF is closely related to Ebola virus disease (EVD).

The DPO and the laboratory technician were part of the taskforce that was set up to control the disease in the 2020 outbreak. However, the area veterinarians were not involved in the prevention and control activities during the outbreak.

The DPO informed the interviewer that there is a disaster preparedness commitment at the district to help in management in disease outbreaks. He highlighted that radio sensitizations are always done in the event of disease outbreaks to inform the public and provide guidelines on how to prevent and control the disease. However, there is a problem of delayed release of funds for these activities. He also mentioned that the district veterinary office is facilitated to conduct regular radio sensitizations to raise awareness about emerging diseases, but this is not done. The slaughterhouse worker, retail meat seller and cattle trader had never heard about the disease.

#### Practices for prevention and control

The laboratory technician, veterinarians and DPO knew about practices for prevention and control of CCHF. The laboratory technician highlighted the following practices for prevention and control of the disease at the hospital setting, wearing personal protective equipment (PPE) for health care workers when handling CCHF-suspected cases and CCHF laboratory samples and proper burial of deceased victims. He also indicated that hospital staff are always sensitized and trained on how to handle suspect cases.

The veterinarians indicated that wearing PPE (like gumboots, overalls and gloves) while performing routine activities like meat inspection, disease diagnosis, pregnancy diagnosis could help to prevent the spread of the virus from animals to humans. However, they highlighted that there are limited resources to buy these consumables, therefore, they rarely use them. They also highlighted that they often sensitize slaughterhouse workers and retail meat sellers about the dangers of zoonotic diseases and how they can prevent them. However, farmers and cattle traders are not trained about zoonotic diseases because this isn’t a priority area for veterinary extension and no funds are allocated for that.

The DPO indicated that prevention and control of emerging infections like CCHF isn’t a priority area for the district. However, in case of an outbreak, there is an emergency fund that is released from the central government to help in disease prevention and control activities. These resources are always limited and therefore, the health sector is prioritized neglecting other important sectors like the environment and veterinary sectors. He also mentioned that one of the major causes of underfunding of CCHF and related diseases is political involvement in the decision making where politically oriented activities are prioritized over prevention and control of emerging diseases. Additionally, periodic surveillance of common diseases is done in the district, however, there is no budget for emerging zoonotic diseases like CCHF. He also mentioned that due to the relatedness of CCHF to Ebola virus disease, the authorities recommend standard operating procedures of Ebola virus disease to manage the outbreak.

There is a common practice of wearing gumboots and overalls among retail meat sellers and abattoir workers, but this is not meant for prevention and control of zoonotic diseases, rather it is for ensuring cleanliness and avoiding injury. They also mentioned that wearing gloves while handling meat would repel potential customers because they would assume that the meat is not safe for human consumption.

#### Epidemiological risk factors

All the participants acknowledged that they are at risk of getting infected with the virus since they are always in close contact with ticks and animals.

Participants who control ticks (3 FGD participants) spray their animals with acaricides monthly but other animals including pigs, sheep and goats are not sprayed with acaricides. One of the participants said,

“*Goats only suffer from internal parasites*.*” (Mr*. *Karema*, *Rutete subcounty)*

## Discussion

This study was aimed at assessing knowledge, attitude, and practices regarding CCHF Kagadi district, located in southwestern Uganda. This is the first study of its kind in Uganda and Africa; it was conducted in the sub counties of Bwikara (where an outbreak was reported) and Ruteete which was randomly selected.

Overall, participants from both sub counties were found to be at high risk for CCHF exposure as most were engaged in animal husbandry activities which increase the risk to CCHF in communities. More specifically, ticks were found to be a common high-risk exposure in the two sub counties as participants reported getting in contact with them during routine activities like grazing animals. This finding is supported by studies by Sharifian et al[9] and Greiner et al[10] which also reported ticks to be a high-risk exposure to community members. Surprisingly, participants reported that they crush ticks using their bare hands. This could be attributed to lack of knowledge about ticks and the threat of tick-borne diseases[11, 12]

Most participants had heard of CCHF before the investigation, which translated into a high knowledge score regarding CCHF.This is in line with findings of Greiner et al[10] which indicate that participants knew about the disease before the study. This could be attributed to the sensitization campaigns conducted in this area after the recent outbreak, therefore residents were more informed about CCHF although gaps in knowledge were still evident. Although most of the participants had heard about the disease, a few had knowledge about it, in regard to transmission, prevention as well as symptoms. This is line with findings of Safieyan et al[9] who reported that participants had no knowledge about disease although they had heard about its existence. The commonest signs and symptoms identified include fever and bleeding. Given the paucity of knowledge on CCHF highlighted earlier, this could be a misconception as these could be related to Ebola virus disease (EVD) which had outbreaks in this region before[13]. During the FGD, participants indicated that CCHF and EVD are closely related, and they were informed by local leaders during the sensitization campaigns that the two diseases present with similar symptoms. This could have biased their reasoning resulting in highlighting EVD symptoms as those of CCHF. A study by Raab et al [14] also acknowledged that knowledge that heightened awareness after the previous EVD outbreak contributed to high knowledge scores of viral hemorrhagic fevers in Guinea.

Although the majority of the participants perceived CCHF to be a fatal disease, study findings revealed that prevention measures were not practiced. This is line with findings of Safieyan et al [9] who reported poor adherence to preventive measures by farmers in Iran although they knew about the disease. Additionally, this is supported by findings of Ziapour et al [15], which reiterate that adhering to preventive behaviors against CCHF is deficient in occupations related to animal husbandry meat handling.

This could be attributed to poor knowledge and attitudes towards the disease. Majority of the participants were farmers with low levels of education, which could have contributed to low knowledge levels. A similar finding was reported by Yilmaz et al[16], thereby indicating that education and occupation are associated with knowledge about CCHF. Failure to practice some preventive measures like wearing PPE can be attributed to high costs of these items and failure of the government to provide them to official veterinarians as tools of service. It was highlighted by the key informants that there are limited logistical resources for One health activities at the local governments. This can be due to low budgetary allocations for sectors involved in One health, that is, health and agriculture [17]. In addition, a study by Beaujean et al [12] indicated that failure to comply with preventive measures like wearing protective clothing is due to climate and belief that wearing this clothing is being overly cautious.

More than a quarter of the participants reported that they slaughter animals at homes without any protective wear like gloves. Additionally, the majority of the respondents reported that their homes are surrounded by bushes, and tick control on the farms is poor. This increases the risk of exposure to the disease. This finding is in line with a study by Ziapour et al [15] who highlighted that individuals involved in livestock management activities practice risky behavior which increases their chances of contracting zoonotic pathogens. Some participants stated that meat consumers perceive that meat handled with gloves isn’t safe, and this misconception promotes neglect of personal protection while handling meat. Such behaviors are practiced regardless of the knowledge of spread of zoonotic pathogens. This is supported by the study conducted in an agropastoral community where agro-pastoralists engaged in risky behaviors, although they knew the existence of a zoonotic disease [18]. They attributed the risky behaviors to culture, social norms, and the belief that the risk is low since they have been practicing those behaviors for generations without any health implication. Additionally, a study by Greiner et al [10] reported that knowledge of CCHF had nothing to do with avoiding risky behaviors. This is attributed to dependence on high-risk activities for livelihood, which is common in impoverished communities. Therefore, risk communication should consider social cultural background of the communities for which the messages are intended [19, 20]. This is because characteristics of the risk (which include severity, prevalence, and perceived effects), social normal and values influence risk assessment and perception by the community [20, 21]. Consequently, this influences the choice of diseases to control and also plays a significant role in individuals’ behavioral change process towards CCHF.

Studies on other zoonoses in Uganda, that is, Marbug and Ebola have highlighted that stigma is one of the biggest issues among survivors in communities after the outbreaks [22]. However, this survey demonstrated that stigma isn’t associated with CCHF. In fact, most participants noted that they would welcome survivors back into the community. This is in line with findings of Raab et al [14]. This attitude could be influenced by community sensitization after the CCHF outbreak.

### Limitations of the study

This study was carried out amidst the COVID19 pandemic, and this had implications on implementation of the study. Initially, the study was designed to conduct two focus group discussions (FGDs), one in each of the sub counties. However, this was not possible because of the fear of the disease. Mobilization of participants was hard due to reluctance to attend gatherings although the district authorities had allowed the study to conduct the FGDs as long as the number of participants in each group wouldn’t exceed 10. We, therefore, decided to conduct one FGD with participants from both sub counties.

Additionally, the researchers had planned to interview three abattoir workers, two policy makers, two veterinarians, three cattle traders and two healthcare workers. However, it was difficult to recruit all these people because of the risk of contracting COVID19 through in-person interactions. The researcher decided to interview one participant from each of these key informant groups (except the veterinarians). The researchers tried to improvise and do phone interviews, but they decided to abandon this method because most of the respondents contacted didn’t answer their phones when called, and those who picked were either occupied with personal tasks or had network challenges. The research team had no prior training on how to conduct phone interviews, and this might have contributed to the failure of this method.

This study employed a cross-sectional design, and thus only collected data to generate informative results. The researchers acknowledge that interventional and promotional studies should have been conducted to improve weak practices of participants in the study area.

## Conclusion

Our study reports low levels of knowledge and awareness on CCHF. There are significant inconsistencies in disease prevention practices among individuals at risk and this highlights a gap in prevention and control of CCHF. A study by Flusin et al [23] recommends that in absence of a CCHF vaccine, prevention of this disease should focus on tick control, personal protection and raising awareness of the population. Therefore, there is a need for educational programs to increase awareness of CCHF in communities and promote CCHF preventive measures. Awareness campaigns have been reported to increase knowledge about viral hemorrhagic fevers after the outbreak [14, 24]. This awareness should be done by both the community leaders and technical people to ensure the community receives accurate knowledge on how to prevent and control the disease. Since the majority of the rural population in Uganda is uneducated and practices farming, educational programs should be targeted at them. This is imperative because the level of knowledge and attitude towards the disease plays a big role in garnering support from the community before any disease control strategy is initiated [25]. Radios are a good medium to spread information about the disease in communities. This was also reported by Nyakarahuka et al [26] where most of the study participants received information about RVF from the radios after the 2016 outbreak in Kabale district. Media is regarded as the greatest source of public knowledge and information regarding infectious diseases like CCHF [27, 28].

In addition to sensitization campaigns on media, awareness campaigns on zoonotic diseases like CCHF can also be integrated into the agriculture extension service program where extensionists who are always in contact with at-risk populations can always sensitize farmers, cattle traders, and abattoir workers on good practices for prevention of CCHF and related diseases. Unfortunately, important actors in prevention and control of CCHF like veterinarians were sidelined during the awareness campaigns conducted after the outbreak, and there is no multidisciplinary coordination at administrative level. Therefore, there is a need to adopt and strengthen a muti-disciplinary approach at all levels in the management of CCHF. This kind of approach is relevant to other communities designing and implementing strategies aimed at prevention and control of CCHF and other emerging and re-emerging zoonotic diseases.

## Supporting information

S1 Questionnaire A questionnaire tool used for data collection. (PDF)
