# Supplementary material for: Knowledge, attitudes, and practices of Crimean Congo hemorrhagic fever among livestock value chain actors in Kagadi district, Uganda
Source: PLoS Negl Trop Dis. 2023 Feb 2;17(2):e0011107. doi: 10.1371/journal.pntd.0011107 (PMC9928074; doi:10.1371/journal.pntd.0011107)
Supplement: S1 Questionnaire — (PDF) [file pntd.0011107.s001.pdf]

Questionnaire tool

CRIMEAN CONGO HEMORRHAGIC FEVER QUESTIONNAIRE *(Tick the one that apply)*

Sociodemographic information

Household number		Date	
Village		Parish	
GPS coordinates			
Sub county		District	
Gender	i) Male…<br>ii) Female….	Age in yrs.	i) 18-30<br>ii) 31-40<br>iii) 41-50<br>iv) 50-60<br>v) More than 60
Level of education	i) None….<br>ii) Primary….<br>iii) Secondary…<br>iv) Tertiary….<br>v) Others specify….		
Major source of livelihood			

10. Diseases in the herd in the *past one-year, have you experienced tick-borne diseases in the herd?*

Disease	Species affected (Cattle, Goats, Dogs)	No. sick	No. died	No. in the flock	Season(s)/month(s) when occurred	V c c (N

If an animal becomes sick, what do you usually do? (tick all that apply)

Do Nothing	Treat it myself (or a family member)	Consult a traditional healer	Consult a community animal health worker	Consult an extension officer	Consult private veterinari

Epidemiological risk factors.

1. Do you have any interaction with ticks during your job?

i) Yes ii) No

2. Do you drink raw milk?

i) Yes ii) No

4. Do you eat raw meat?

i)Yes ii) No

5. Are there bushes around the farm?

i) Yes ii) No

6. ***Production systems***

Species	7. Production type: *what is the main reason for keeping each species?*			8. Production system: *what is the m* *system?*		
	Dairy	Meat	Multi-purpose	Sedentary mixed farming	Agro-pastoralist	
Cattle						
Goats/sheep						

7. *Proposing factors to CCHF*

11. Are there wild animals like antelopes, warthogs or others specify that come into your farm?	

12. Do you see any vermins on the farm e.g. rodents, squirrels, bats, wild birds?	
13. How do you regularly clear your farmland?	
14. Do you slaughter or butcher or assist with butchering livestock?	
15 Does the subject attend to livestock management activities?	
Does the respondent crush ticks with bare hands?	
16.Others, please specify………………………………………………………………………………………	

Practices for prevention and control.

1. What do you do to protect yourself from ticks/CCHF?

a. use protective clothing (i.e. long pants, socks, etc.) i) Yes…. ii) No….

How often? i) Always…… ii) Sometimes iii) Never

b. Treat your clothing with repellent

i) Yes …… ii) No……

How often? i) Always ii) Sometimes iii) Never

c. use insect repellent on yourself i) Yes …… ii) No……

How often? i) Always ii) Sometimes iii) Never

d. Use pesticides in the environment i) Yes …… ii) No……

How often? i) Always ii) Sometimes iii) Never

e. Avoid woody/rural areas i) Yes …… ii) No……

How often? i) Always ii) Sometimes iii) Never

h. Other_________________

2. Do you control ticks on the farm?

i) Yes ii) No

If yes?

A) Which method?

a) Spraying Dipping b) Hand picking c) Bush burning in dry season d) Others, specify……

B) How often?

a) Once a week b) Once in 2 weeks c) Once a month d)Others, specify……

C) Is tick control done in other animals?

a) Yes b) No

If yes, which ones?

b) goats b) sheep c) pigs d) dogs e) others, specify…..

Do you wear protective clothing while spraying animals?

i) Yes ii) No

3. What care would you seek, if any, if you experienced symptoms of CCHF?

i) Go to a hospital/healthcare ii) Stay at home iii) Try local pharmacy iv) Go to a local healer v) Other, specify

Knowledge & Attitude questions

1. Have you heard of CCHF?

Yes ii) No If yes, from who?

i) Health worker ii) Radio iii) community leaders iv) others, specify……

2. Do you know the signs and symptoms of CCHF in humans?

i) Yes ii) No

If yes, what are some signs and symptoms?

ii) Bleeding ii) fever iii) diarrhoea

3. Do you know who to contact in case you see the signs and symptoms?

i) Yes ii) No 4. Do you believe CCHF really exists?

i) Yes ii) No If no, why?..

4. Have you heard of any survivor of CCHF?

i) Yes ii) No

5. Would you relate or interact with the survivor of CCHF?

i) Yes ii) No

6. Would you welcome CCHF survivor back into the community?

i) Yes ii) No

If no, why?....................................

7. Do you know how CCHF is transmitted?

i) Yes ii) No

If Yes, how?

i) Tick bite ii) contact with blood and other tissues iii) others, specify….

If transmission from animals, which ones?

iii) Sheep ii) cattle iii) goats iv) dogs v) poultry vi) others, specify……

8. How do you think you can protect yourself from acquiring CCHF?

i) Avoiding contact with ticks ii) avoiding contact with animals iii) others, specify……….

9. How do you think CCHF can best be healed?

i) Modern medicine ii) traditional medicine iii) herbal medicine iv) spiritual healing v) others, specify……………

10. Do you think you are at risk of contracting CCHF?

i) Yes ii) No

Why?..

11. Do you know how CCHF can be prevented?

i) Yes ii) No

If yes, how?....................................

……………………………………………………….

Thank you for your time.
